# Activation of the mTOR Pathway by Oxaliplatin in the Treatment of Colorectal Cancer Liver Metastasis

**DOI:** 10.1371/journal.pone.0169439

**Published:** 2017-01-06

**Authors:** Min Lu, Amelia S. Zessin, Wayne Glover, David S. Hsu

**Affiliations:** 1 Department of Medical Oncology, Duke University Medical Center, Durham, North Carolina, United States of America; 2 Center for Genomics and Computational Biology, Duke University, Durham, North Carolina, United States of America; University of Navarra, SPAIN

## Abstract

**Background:**

Standard of care treatment for colorectal cancer liver metastasis consists of a cytotoxic chemotherapy in combination with a targeted agent. Clinical trials have guided the use of these combinatory therapies, but it remains unclear what the optimal combinations of cytotoxic chemotherapy with a targeted agent are.

**Methods:**

Using a genomic based approach, gene expression profiling was obtained from tumor samples of patient with colorectal cancer liver metastasis who received an oxaliplatin based therapy. Early passaged colorectal cancer liver metastasis cell lines and patient derived xenografts of colorectal cancer liver metastasis were then treated with oxaliplatin and a mTOR inhibitor.

**Results:**

Gene set enrichment analysis revealed that the mTOR pathway was activated in patients receiving oxaliplatin based therapy. Treatment of early passaged colorectal cancer lines and patient derived xenografts with oxaliplatin resulted in activation of the mTOR pathway. Combination therapy with oxaliplatin and a mTOR inhibitor resulted in a synergistic effect both *in vitro* and *in vivo*.

**Conclusion:**

Our findings suggest a genomic based approach can be used to identify optimal combinations of cytotoxic chemotherapy with a targeted agent and that these observations can be validated both *in vitro* and *in vivo* using patient derived colorectal cancer cell lines and patient derived xenografts prior to clinical use.

## Background

Chemotherapy has been used as the backbone for the treatment of cancer for the past few decades. Currently, the choice of treatment for an individual patient with metastatic colorectal cancer is based on patient and physician preference and empiric clinical trial data. Standard of care treatment of metastatic colorectal cancer consists of cytotoxic chemotherapy in combination with a targeted agent. Specifically, 5- fluorouracil (5-FU) is combined with either oxaliplatin (FOLFOX) or irinotecan (FOLFIRI) and then with either a VEGF (vascular endothelial growth factor) inhibitor such as bevacizumab or an EGFR (epithelial growth factor receptor) inhibitor such as cetuximab or panitumumab. However, response rates when either of these two targeted agents is combined with cytotoxic chemotherapy are still only 60–65% [1–3] suggesting that other combinations need to be identified and tested to achieve maximal response and benefit.

Therapy based upon the biology of an individual’s tumor rather than established histopathological and anatomical classification can optimize the use of existing therapy and identify new and novel targets. As genetic and epigenetic events that results in cancer are discovered, treatment based on the biology of the cancer are being developed [4–6]. Specifically, models of colorectal cancer based on genomic technologies have increased our molecular understanding of colorectal cancer. Using these models, the identification of key molecular events driving colorectal cancer initiation, progression, and metastasis are being used to individualized therapies. [4, 7]

However, prior to the incorporation of new therapeutic agents in the clinical setting, these drugs must be assessed for their therapeutic potential in predictive preclinical models, and therefore, mouse xenografts have been developed to screen new cancer drugs.[8] Initially, athymic mice (nu/nu) and SCID mice were used to establish xenografts from human tumor cell lines to test their response to cancer drugs.[9] In contrast, recently we and others have demonstrated that an approach of rapid engraftment of patient tumor into immunodeficient mice to develop patient derived xenografts (PDXs) which may provide a more clinically applicable murine model to study drug sensitivities.[10–15] Specifically, we have shown that the biology of PDXs is similar to the corresponding patient tumors, at both the histologic and molecular levels after multiple passages.[15, 16] Furthermore, early passaged cell lines can be easily grown from the corresponding PDXs and thus the combination of early passage cell lines and PDXs provide powerful preclinical models to test new therapeutics.

In our present work, we use a combination of a genomic based approach with preclinical models of colorectal cancer to identify synergistic drugs that can be used for the treatment of colorectal liver metastasis. First, a gene expression profiling was performed to identify signaling pathways in colorectal cancer liver metastasis that become activated upon treatment with oxaliplatin. Based on these methods, we identified the mTOR pathway as an oxaliplatin induced signaling pathway. Next, we showed that a combination of oxaliplatin and mTOR inhibition (rapamycin) has a synergistic effect *in vitro*. Finally, these findings were then validated *in vivo* in a patient derived xenograft (PDX) model of colorectal cancer. Everolimus was used in PDX model because it is a FDA proved orally administered rapamycin analog and it has superior pharmaceutical characteristics to rapamycin [17]. Our results suggest that the combination of oxaliplatin and mTOR inhibition can be used effectively to treat patients with colorectal cancer liver metastasis.

## Materials and Methods

### Gene expression analysis

Colorectal cancer liver metastasis specimens were collected under a Duke IRB approved protocol (Pro00002435). Tumor specimens collected were frozen in OCT (Optimal Cutting Temperature). An initial section of each OCT block was stained using H&E (Hematoxylin and Eosin) to determine tumor percentage and necrosis. Macrodissection of the tumor was then performed to ensure >70% tumor content. RNA was isolated from tumor specimen using a Qiagen RNA/DNA Allprep kit, converted to cDNA and labeled by one cycle IVT. IVT labeled cDNAs were prepared according to the manufacturer’s instructions, and targets hybridized to the Human U133A 2.0 GeneChip and read on an Affymetrix array scanner. Raw data was converted to CEL files and RMA normalized. CEL files used in this manuscript are available at the Gene Expression Omnibus (GEO) (http://www.ncbi.nlm.nih.gov/geo/) (GSE41568).

GenePattern (http://www.broad.mit/edu/cancer/software/genepattern) was used to perform both unsupervised and supervised analysis. To check for sample outliers and batch effects, 3D principal components analysis of the global gene expression was performed. Data was first preprocessed to 8263 (min fold = 7, min delta = 5), 6714 genes (min fold = 8, min delta = 5) and 3307 genes (min fold = 13, min delta = 5) Hierarchical clustering was performed using complete linkage with a Pearson correlation metric method on the preprocessed data; samples were clustered into a single tree.[18] To determine pathways associated with oxaliplatin, gene set enrichment analysis (GSEA). Gene sets were first preprocessed to exclude gene sets with <10 and >500 genes. Ten thousand iterations were then performed per analysis with a signal to noise metric used to rank genes based upon their differential expression across the two classes. For discovery, gene sets with a normalized p-value < 0.05 were identified.

### Colorectal Cancer Cell Line and Patient Derived Xenograft Development

Patient specimens of CRC liver metastasis were collected under a Duke IRB approved protocol (Pro00002435). All participants provided written informed consent to participate in the study. Patient derived xenografts (PDXs) of colorectal cancer liver metastasis were then developed as described previously [15, 16] and all mouse experiments were carried out in accordance with the animal guidelines and with the approval of the Institutional Animal Care and Use Committee (IACUC) at the Duke University Medical Center." Briefly, to develop PDXs, the tissues were washed with phosphate buffered saline (PBS) and then minced into pieces approximately ~2 mm in size and injected into the flanks of 8-10-week-old JAX NOD.CB17-PrkdcSCID-J mice obtained from Duke University Rodent Genetic and breeding core. Monitoring for tumor burden was performed and mice were euthanized when the tumors reach a size of >2000 mm3, become ulcerated, the mice pay undue attention to or chew on the lesion, interfere with 'normal' mouse functions (e.g. eat, drink, or ambulate) or tumor burden is greater than 1% of the baseline body weight. At this time, mice were euthanized via CO_2_ inhalation chamber and this method was chosen because it is consistent with the recommendations of the American Veterinary Medical Association Panel on Euthanasia and is approved by the Duke University IACUC. During our experiments, no mice became severely ill or died prior to the experimental endpoint.

Early passage colorectal cancer liver metastasis cell lines were developed from the PDXs as followed. PDXs were harvested, homogenized and grown in cell culture media (RPMI 1640 media, 10% fetal calf serum, 10 U/ml penicillin and streptomycin) at 5% CO_2_. Clonal population of cell line was then obtained by isolating a single clone using an O ring. The following CRC PDX and cell lines were generated and used in this study; CRC057, CRC119 and CRC240. Cell lines were authenticated using the Duke University DNA Analysis Facility Human cell line authentication (CLA) service by analyzing DNA samples from each individual cell lines for polymorphic short tandem repeat (STR) markers using the GenePrint 10 kit from Promega (Madison, WI, USA).

HCT15, DLD-1, LoVo, HT29 and Colo205 colorectal cancer cell lines were obtained from ATCC (American Type Culture Collection) and were not authenticated as they were used < 6 months from purchase date.

### *In vitro* Drug Sensitivity Assays

CRC057, CRC119, CRC240, HCT15, DLD-1, LoVo, HT29 and Colo205 were grown in RPMI 1640 media + 10% FBS and plated in drug-free medium at a concentration of 5000 cell/well in tissue culture treated 96 well plate. Five replicate wells were used for each planned drug concentration. Control cells included cells plated in growth media without drugs and wells with growth medium but without cells. After 24 hrs of incubation at 37°C, each cell line was exposed to a series of increasing drug concentration (0.0001-1000uM for oxaliplatin and 0.0016-125uM for rapamycin) and subsequently incubated at 37°C for 4d. Cell cytotoxicity was assayed with CellTiter-Glo® Luminescent Cell Viability Assay kit (Promega, USA) at day 4. Corresponding EC50 for oxaliplatin and rapamycin was calculated for each cell line using the GraphPad Prism software (La Jolla, CA, USA).

Synergy for the combination of targeted pathway inhibition with conventional cytotoxic therapy was assessed using the methods of Chou and Talalay within the Calcusyn software (Biosoft, Inc., United Kingdom)[19]. Briefly, serial dilutions of oxaliplatin (0.002 uM-1uM) and rapamycin (0.2 uM-35 uM) were applied to colorectal cancer cell lines as a fixed-ratio serial dilution of oxaliplatin-EC50: rapamycin-EC50 of each cell lines (CRC057, CRC119, CRC240, HCT15, DLD-1, LoVo, HT29 and Colo205). Oxaliplatin was obtained from the Duke University Pharmacy Stockroom and rapamycin was purchased from Sigma-Aldrich (St. Louis, MO). Cytotoxicity was assessed at day 4 as described previously.[20] A combination index (CI) was calculated based on the Chou and Talalay method using the Calcusyn software (Biosoft, Cambridge, UK) with a Combination Index (CI) <1 implying synergy, >1 antagonism, and equal to 1 additive [19]. Experiments were performed in triplicate with mean CI and standard deviation (SD) calculated.

### *In vivo* Drug Sensitivity Assays

To test the sensitivity of CRC057, CRC119 and CRC240 PDX to oxaliplatin and everolimus, CRC057, CRC119 and CRC240 PDX tissue were minced in PBS at 150 mg/ml. 200ul of each PDX tissue suspension were subcutaneously injected into the flanks of JAX NOD.CB17-PrkdcSCID-J mice (10 week old male mice). When tumor volume reached approximately 200 mm^3^, mice were randomized and treated either with oxaliplatin (10 mg/kg or 5 mg/kg) and/or everolimus (5 mg/kg or 2.5 mg/kg, obtained from Duke University Pharmacy Stockroom). Oxaliplatin was administered weekly via intraperitoneal injection for 3 weeks, and saline was used as a control. Everolimus was administered by oral gavage in 100 μL of Ora-Plus suspending vehicle (Perrigo, Allegan, MI) every other day for 3 weeks, and vehicle alone was used as a control. Each group consisted of 6 or 7 mice. Tumor size was measured at least twice a week with a vernier caliper, and tumor volume was calculated as follows until humane endpoint (> 2000 mm^3^). Tumor volume = (width)^2^ x length/2. All mouse experiments were carried out in accordance with the animal guidelines and with the approval of the Institutional Animal Care and Use Committee (IACUC) at the Duke University Medical Center.

### Western Blotting

The cells and homogenized PDX tissue were lysed in RIPA buffer supplied with Protease inhibitor and Phosphatase inhibitor (Thermo Scientific Inc., USA). Western blotting was carried out as the standard protocol and as followed. Total protein concentrations were quantified using DC protein Assay (Bio-rad, USA). Proteins were separated by SDS-PAGE Gel Electrophoresis and subsequently transferred onto polyvinylidene fluoride (PVDF) membranes. PVDF membranes were blocked with nonfat milk for 1 hour at room temperature. Primary antibodies (p70 S6 Kinase (49D7), Phospho- p70 S6 Kinase (Thr389), and β-actin) (Cell signaling, USA) were added according to the specifications of the manufacturer's protocols. Corresponding Horse Radish Peroxidase (HRP) conjugated secondary antibodies were added at 1:10000 dilution for 1 hour at room temperature. Membranes were washed with Tris Buffered Saline + Tween-20 (TBST). Membranes were exposed upon addition of chemiluminescent reagent using X-ray film.

### Statistical Analysis

PDX tumor sizes were recorded in GraphPad Prism 6 software (La Jolla, CA, USA) and One-way ANOVA analysis was used to compare the tumor size between groups (p < 0.05 was considered to have statistical significance).

## Results

### Gene Expression Profiling to Identify Upregulated Signaling Pathway in Oxaliplatin treated Colorectal Cancer

**Fig 1:**
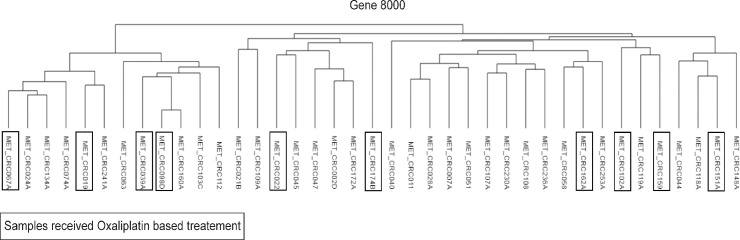
Unsupervised hierarchical clustering on 39 colorectal cancer liver metastasis samples were performed with oxaliplatin treated samples boxed. Unsupervised hierarchical clustering showed that oxaliplatin treatment did not influence the clustering of the 39 samples.

In order to identify cancer related signaling pathways that were upregulated in colorectal cancer patients treated with oxaliplatin based therapy, gene expression data using the Affymetrix U133 array was obtained on 39 patients who underwent resection of their colorectal cancer liver metastasis at Duke University between 2007 and 2011. There were 21 males and 18 females in this cohort and the majority of these patients were Caucasian (31/38). 11 of these patients received an oxaliplatin based therapy prior to their liver resection while 28 received no treatment prior to surgery (**Table 1**). All patients received at least 3 months of therapy and underwent resection of their cancer within 4–6 weeks after their last oxaliplatin dose. We first performed an unsupervised hierarchical clustering on the data set using a preprocessed data set of approximately 8000 genes and **Fig 1** showed that treatment with an oxaliplatin based therapy did not influence the clustering of the samples. Similar results were observed using 3000 and 6000 genes (**S1 Fig**).

**Table 1:** Clinical characteristics of 39 colorectal cancer patients with liver metastasis.

Sample ID	Primary Site	Metastatic Site	Gender	Race	Neoadjuvant Chemo prior to liver resection
CRC019	Colon	Liver	Male	W	FOLFOX
CRC022	Colon	Liver	Male	W	FOLFOX
CRC039	Colon	Liver	Male	W	FOLFOX
CRC067	Colon	Liver	Female	W	FOLFOX + Bevacizumab
CRC098	Colon	Liver	Female	W	XELOX + Bevacizumab
CRC102	Colon	Liver	Female	AA	XELOX + Bevacizumab
CRC119	Colon	Liver	Female	AA	FOLFOX
CRC151	Colon	Liver	Female	W	FOLFOX
CRC159	Colon	Liver	Male	W	FOLFOX
CRC162	Colon	Liver	Female	AA	XELOX + Bevacizumab
CRC174	Colon	Liver	Female	AA	XELOX + Bevacizumab
CRC002	Colon	Liver	Female	W	none
CRC007	Colon	Liver	Male	W	none
CRC011	Colon	Liver	Male	W	none
CRC021	Colon	Liver	Male	W	none
CRC024	Colon	Liver	Female	W	none
CRC028	Colon	Liver	Female	W	none
CRC040	Colon	Liver	Female	W	none
CRC044	Colon	Liver	Female	W	none
CRC045	Colon	Liver	Male	W	none
CRC047	Colon	Liver	Male	AA	none
CRC051	Colon	Liver	Male	W	none
CRC058	Colon	Liver	Male	W	none
CRC063	Colon	Liver	Male	W	none
CRC074	Rectal	Liver	Female	W	none
CRC103	Colon	Liver	Female	Indian	none
CRC107	Colon	Liver	Male	W	none
CRC108	Colon	Liver	Male	AA	none
CRC109	Colon	Liver	Female	W	none
CRC112	Colon	Liver	Male	W	none
CRC118	Colon	Liver	Female	W	none
CRC134	Colon	Liver	Male	AA	none
CRC148	Colon	Liver	Female	W	none
CRC160	Colon	Liver	Male	W	none
CRC172	Colon	Liver	Male	W	none
CRC230	Colon	Liver	Male	W	none
CRC236	Colon	Liver	Male	W	none
CRC241	Colon	Liver	Female	W	none
CRC253	Colon	Liver	Male	W	none

We next performed a supervised analysis using GSEA [21] algorithm using the C6 (oncogenic signatures) gene sets to identify differentially regulated oncogenic signaling pathways in the oxaliplatin treated samples. Using a discovery-focused statistical threshold (p < 0.05), 21 gene sets were found to be enriched/upregulated in colorectal cancer liver metastasis tumors treated with oxaliplatin. These pathways were found to be involved in cellular differentiation, cell cycle, signaling pathways, epigenetic, metabolism and immunology (**Table 2 and S1 Table**). As we were interested in identifying targets that could be synergistic with oxaliplatin, we looked in the signaling pathway group for pathways that could be targeted and identified the WNT/β-catenin, RAS, PI3Kinase, MAP Kinase and the mTOR pathway. Based on our interest in the mTOR pathway, we decided to further study the interaction between oxaliplatin and the mTOR pathway.

**Table 2:** Enriched/upregulated oncogenic signaling pathways in patients with colorectal cancer liver metastasis treated with oxaliplatin.

	Description	Reference
**Cellular differentiation**		
ESC_J1_UP_LATE.V1_DN	Genes down-regulated during late stages of differentiation of embryoid bodies from J1 embryonic stem cells	http://software.broadinstitute.org/gsea/msigdb/cards/ESC_J1_UP_LATE.V1_DN
GCNP_SHH_UP_LATE.V1_DN	Sonic hedgehog (SHH) is one of three proteins in the mammalian signaling pathway family called hedgehog	http://software.broadinstitute.org/gsea/msigdb/cards/GCNP_SHH_UP_LATE.V1_DN.html
CRX_NRL_DN.V1_UP	Cone-rod homeobox protein (CRX) is a photoreceptor-specific transcription factor which plays a role in the differentiation of photoreceptor cells	http://software.broadinstitute.org/gsea/msigdb/cards/CRX_NRL_DN.V1_UP
ATF2_UP.V1_DN	Activating transcription factor 2 (AFT2) is a member of the leucine zipper family of DNA-binding proteins	http://software.broadinstitute.org/gsea/msigdb/cards/ATF2_UP.V1_DN.html
E2F1_UP.V1_DN	Transcription factor E2F1 is a member of the E2F family of transcription factors. The E2F family plays a crucial role in the control of cell cycle and action of tumor suppressor proteins	http://software.broadinstitute.org/gsea/msigdb/cards/E2F1_UP.V1_DN
CRX_DN.V1_DN	Cone-rod homeobox protein (CRX) is a photoreceptor-specific transcription factor which plays a role in the differentiation of photoreceptor cells	http://software.broadinstitute.org/gsea/msigdb/cards/CRX_DN.V1_DN
ATF2_S_UP.V1_DN	Abnormal activation of Activating transcription factor 2 (ATF2) in growth and progression of mammalian skin tumors	http://software.broadinstitute.org/gsea/msigdb/cards/ATF2_S_UP.V1_DN
NRL_DN.V1_UP	Neural retina-specific leucine zipper protein (NRL) is a transcription factor	http://software.broadinstitute.org/gsea/msigdb/cards/NRL_DN.V1_UP
**Cell cycle**		
CYCLIN_D1_KE_.V1_UP	Cyclin D1 is a protein required for progression through the G1 phase of the cell cycle	http://software.broadinstitute.org/gsea/msigdb/cards/CYCLIN_D1_KE_.V1_UP
**Signaling pathways**		
BCAT.100_UP.V1_DN	β-catenin is a dual function protein, regulating the coordination of cell–cell adhesion and gene transcription	http://software.broadinstitute.org/gsea/msigdb/cards/BCAT.100_UP.V1_DN
KRAS.DF.V1_DN	KRAS performs an essential function in normal tissue signaling, and the mutation of a KRAS gene is an essential step in the development of many cancers	http://software.broadinstitute.org/gsea/msigdb/cards/KRAS.DF.V1_DN
PKCA_DN.V1_DN	PKCα. PKC family members serve as major receptors for phorbol esters, a class of tumor promoters	http://software.broadinstitute.org/gsea/msigdb/cards/PKCA_DN.V1_DN
PTEN_DN.V2_DN	Phosphatase and tensin homolog (PTEN) is a protein that, in humans, is encoded by the PTEN gene. Mutations of this gene are a step in the development of many cancers	http://software.broadinstitute.org/gsea/msigdb/cards/PTEN_DN.V2_DN
JNK_DN.V1_DN	JNK plays a role in T cell differentiation and the cellular apoptosis pathway	http://software.broadinstitute.org/gsea/msigdb/cards/JNK_DN.V1_DN
WNT_UP.V1_UP	The conserved Wnt/β-Catenin pathway regulates stem cell pluripotency and cell fate decisions during development	http://software.broadinstitute.org/gsea/msigdb/cards/WNT_UP.V1_UP
MTOR_UP.N4.V1_DN	The PI3K/AKT/mTOR pathway is an intracellular signaling pathway important in regulating the cell cycle	http://software.broadinstitute.org/gsea/msigdb/cards/MTOR_UP.N4.V1_DN
KRAS.AMP.LUNG_UP.V1_UP	KRAS. it performs an essential function in normal tissue signaling, and the mutation of a KRAS gene is an essential step in the development of many cancers	http://software.broadinstitute.org/gsea/msigdb/cards/KRAS.AMP.LUNG_UP.V1_UP
BCAT_GDS748_UP	β-catenin is a dual function protein, regulating the coordination of cell–cell adhesion and gene transcription	http://software.broadinstitute.org/gsea/msigdb/cards/BCAT_GDS748_UP
**Epigenetics**		
SNF5_DN.V1_DN	SNF5. Involved in transcriptional activation	http://software.broadinstitute.org/gsea/msigdb/cards/SNF5_DN.V1_DN
**Metabolism**		
DCA_UP.V1_DN	Dichloroacetic acid (DCA), inhibit the enzyme pyruvate dehydrogenase kinase	http://software.broadinstitute.org/gsea/msigdb/cards/DCA_UP.V1_DN
**Immunology**		
HINATA_NFKB_IMMU_INF	NF-κB is a protein complex that controls transcription of DNA, cytokine production and cell survival	http://software.broadinstitute.org/gsea/msigdb/cards/HINATA_NFKB_IMMU_INF

### Oxaliplatin Induced mTOR Activation *in vitro*

**Fig 2:**
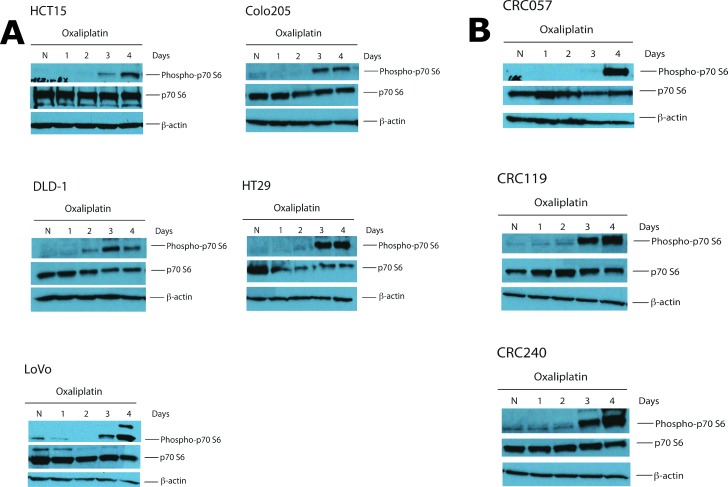
Activation of the mTOR pathway by oxaliplatin *in vitro*. **A.** The level of phosphorylated p70 S6 kinase was increased in all five ATCC cell lines (HCT15, DLD-1, LoVo, HCT116, HT29 and Colo 205) by Day 3. **B.** The level of phosphorylated p70 S6 kinase was increased in all early passage colorectal cancer cell lines (CRC057, CRC119 and CRC240) by Day 3 or 4.

We first wanted to determine if treatment of CRC cell lines with oxaliplatin activates the mTOR pathway. Five colorectal cancer cell lines obtained from ATCC (American Type Culture Collection; HCT15, DLD-1, HT29, LoVo and Colo205) were treated with oxaliplatin and rapamycin and the IC50s to oxaliplatin and rapamycin for these cell lines were determined (**Table 3**). Each cell line was then treated with oxaliplatin at the IC50 dose for 1–4 days. At baseline (Day 0), there was no activation of the mTOR pathway based on levels of phosphorylated p70 S6. However, by Day 3, all six cell lines displayed activation of the mTOR pathway (**Fig 2A**). To further verify this observation, we performed the same experiment on 3 early passaged colorectal cancer cell lines that were developed from tumors of patients undergoing resection of the colorectal cancer liver metastasis (CRC057, CRC119 and CR240). Similar to the ATCC CRC cell lines, at the IC50 dose, activation of the mTOR pathway was observed by Day 3 in our early passaged CRC cell lines (**Fig 2B**).

**Table 3:** IC50 of oxaliplatin and rapamycin in 3 early passage colorectal cancer cell lines and 5 ATCC cell lines.

	Cells	Oxaliplatin IC50	Rapamycin IC50	Fixed radio (Oxaliplatin IC50: Rapamycin IC50)
CRC cell lines	CRC057	0.18uM	17.54uM	1:93.6
	CRC119	0.6uM	22.35uM	1:37.5
	CRC240	0.53uM	37.37uM	1:70.5
ATCC cell lines	HCT15	3uM	21uM	1:7
	DLD-1	6uM	24uM	1:4
	LOVO	0.5uM	20uM	1:40
	HT29	2uM	16uM	1:8
	COLO205	2uM	32uM	1:16

### Synergy Between Oxaliplatin and Rapamycin *in vitro*

**Fig 3:**
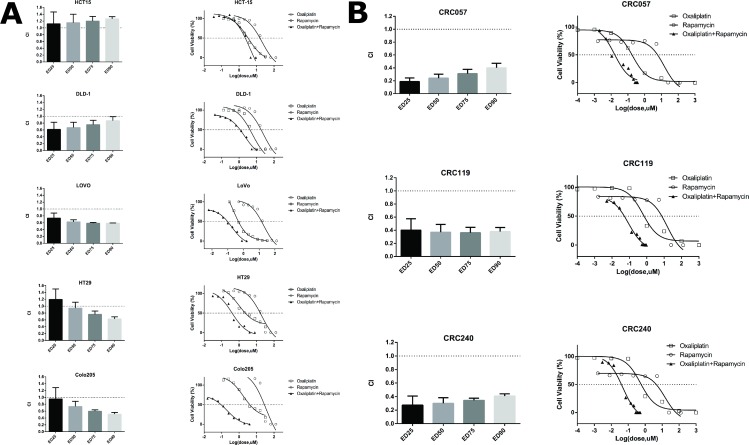
Oxaliplatin and Rapamycin Synergy Graphs were performed to determine the combination index (CI). **A**. The growth of ATCC colorectal cell lines HCT15, DLD-1, LoVo, HCT116, HT29 and Colo 205 in the presence of oxaliplatin and rapamycin as single agents and in combination was analyzed by cytotoxicity assays (see materials and methods) to determine the dual-effect of the two agents on the cell lines. Mean Combination Index (CI) values (from three experiments) at 25%, 50%, 75% and 90% is plotted for each cell line. (CI < 1 denotes synergy between the two drugs). Oxaliplatin and rapamycin did show synergistic effect in DLD-1, LoVo, HT29 and Colo205 cell lines except HCT15 cell line. **B**. The growth of early passage colorectal cancer cell lines CRC057, CRC119 and CRC240 in the presence of oxaliplatin and rapamycin as single agents and in combination was analyzed by cytotoxicity assays (see materials and methods) to determine the dual-effect of the two agents on the cell lines. Mean Combination Index (CI) values (from three experiments) at 25%, 50%, 75% and 90% is plotted for each cell line. (CI < 1 denotes synergy between the two drugs). Oxaliplatin and rapamycin had synergistic effect in all three cell lines.

As oxaliplatin activated the mTOR pathway, we next wanted to determine if the combination of oxaliplatin and a mTOR inhibitor was synergistic. The five ATCC cell lines (HCT15, DLD-1, LoVo, HT29 and Colo205) were treated with either oxaliplatin (IC50 dose), rapamycin (IC50 dose) or in combination at a fixed ratio (**Table 3**) for 4 days The combination index (CI) of these two drugs in each cell lines was calculated using the Calcusyn software at the ED25, ED50 and ED75 and ED90 to determine synergy based on the Chou and Talalay method.[19] **Fig 3A** show that no synergy was observed between oxaliplatin and rapamycin in HCT15 (CI > 1). In DLD-1 and LoVo, synergy was observed between oxaliplatin and rapamycin (CI < 1); in Colo205, synergy was observed at ED50, ED75 and ED90; and in HT29, synergy was observed at ED75 and ED90. To further verify this observation, this experiment was performed in our 3 early passaged colorectal cancer cell lines (CRC057, CRC119 and CRC240). Similar to the ATCC cell lines, synergy was also observed between oxaliplatin and rapamycin in all three of our early passaged colorectal cancer cell lines (CI < 1) (**Fig 3B**).

### Oxaliplatin Induced mTOR Activation *in vivo*

**Fig 4:**
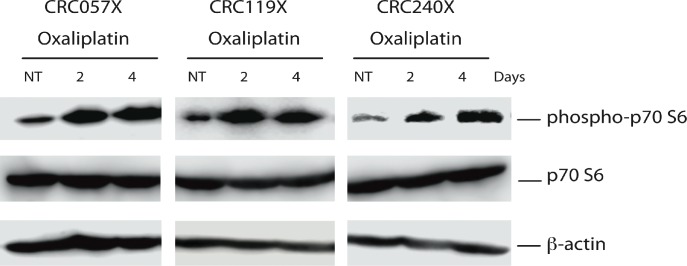
Activation of the mTOR pathway by oxaliplatin *in vivo*. The level of phosphorylated p70 S6 kinase was increased in colorectal cancer PDXs by Day 2.

To determine if the effects observed *in vitro* were also observed *in vivo*, we used our patient derived xenograft (PDX) model of colorectal cancer. CRC119 was established subcutaneously in the flanks of JAX NOD.CB17-PrkdcSCID-J mice as previously described.[15, 16] When the tumor volume reached approximately 200 mm^3^, mice were treated with oxaliplatin (10 mg/kg, Day 0) and tumors were harvested on Days 2 and 4. Western blots showed that similar to the *in vitro* experiments with the cell lines, the level of phospho-p70 S6 kinase was increased during the oxaliplatin treatment period from the second day to the fourth day in our CRC119 PDX model (**Fig 4**).

### Synergy between Oxaliplatin and Everolimus *in vivo*

**Fig 5:**
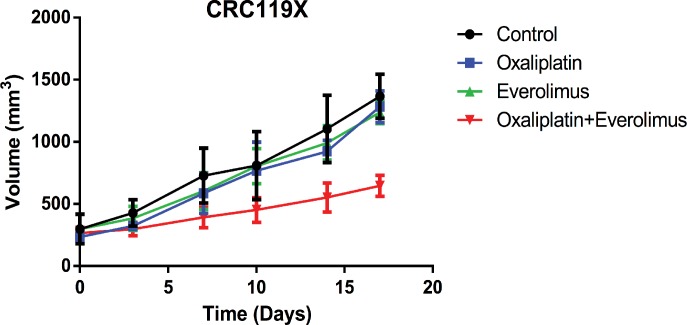
Synergy between oxaliplatin and everolimus *in vivo*. Oxaliplatin and everolimus were shown to have synergic effect in CRC119 PDX based on tumor growth inhibition with the combination therapy compared to either oxaliplatin or everolimus alone. * Tumor sizes at Day 21, Control vs. Oxaliplatin + Everolimus: P<0.05.

Finally, to determine if the synergistic effect observed *in vitro* was also observed *in vivo*, synergy experiments were performed. CRC119 was established subcutaneously in the flanks of JAX NOD.CB17-PrkdcSCID-J mice and when the tumor volume reached approximately 200 mm^3^, mice were treated with oxaliplatin (10 mg/kg IP weekly), everolimus (5 mg/kg oral gavage every other day) or in combination (oxaliplatin 10 mg/kg IP weekly and everolimus 5 mg/kg oral gavage every other day). **Fig 5** shows that CRC119 was resistant to both everolimus and oxaliplatin (p > 0.05). However, the combination of everolimus and oxaliplatin resulted in significance decrease in tumor growth inhibition (p < 0.05) suggesting a synergistic effect between the two drugs.

## Discussion

Currently, the treatment of colorectal cancer in the first line setting using targeted therapy is limited to anti-VEGF therapy (bevacizumab, aflibercept) or anti-EGFR therapy (cetuximab, panitumumab) in combination with an oxaliplatin based therapy. [1–3] However, it remains unclear whether other signaling pathways can be used in combination with oxaliplatin to improve clinical outcomes. Thus, genomic-based strategies are now being used to model colorectal cancer [22–24] to better understand the biology of the disease and to develop better therapeutic options. Specifically, gene expression profiling can measure genome-wide gene expression activity that can be used to identify discrete biologically relevant phenotypes to characterize a disease.[18, 25] This can then be used to characterize phenotypes such as response to drugs to help guide existing therapies and discover new therapeutic targets.[26–28] Thus, in our current work, we used gene expression profiling to identify signaling pathways that are activated upon treatment with oxaliplatin in colorectal cancer and discovered that the mTOR pathway was one such pathway.

mTOR is a serine/threonine protein kinase that regulates cell growth, cell proliferation, cell motility, cell survival, protein synthesis, and transcription and has been studied in colorectal cancer a potential therapeutic target. One potential agent to target mTOR in colorectal cancer is everolimus (RAD001) and there have been several early studies of the mTOR inhibitors suggesting some benefit in these patients. In one phase I trial of RAD-001, one partial response (PR) was seen in a patient with colorectal cancer lasting 5.3 months with a disease free survival (DFS) of 9 months.[29] In the subsequent phase II study, a disease control rate of 25%, with an OS of 5.9 months was achieved. However, to date, the benefits of everolimus in colorectal cancer have been minimal and thus suggest that monotherapy with a mTOR inhibitor may not be clinically efficacious. In contrast, mTOR inhibitors appear to be more effective when combined with cytotoxic chemotherapy and in one phase I clinical trial, the combination of RAD001 with 5-fluorouracil in a refractory colorectal cancer patient population resulted in one PR lasting 7.4 months.[30]

Thus, our work is the first study to determine the effectiveness of combining everolimus and oxaliplatin using early-passaged cell lines derived from colorectal cancer liver metastasis tumors and colorectal cancer liver metastatic patient-derived xenograft (PDX) model. Previous studies tested the combination of everolimus and cisplatin in non-small cell lung cancer (NSLC) in mice xenografts models and observed synergy with the combination in NSCLC. [31] As cisplatin is not used in colorectal cancer, our data with oxaliplatin suggests that synergy with everolimus can also be observed with other platinum agents. Another study showed that the combination of mTOR inhibitor with 5-FU, oxaliplatin and SN38 in colorectal cancer cell lines had synergistic effect only in the cell lines which contained a PI3K or P53 mutation with wide-type KRAS. [32] In contrast, CRC057 was found to have mutations in KRAS, PI3Kinase and APC, CRC119 had a mutation only in KRAS and CRC240 has mutations in NRAS and p53 (data not shown). Although a small data set, our results suggest that mutations in PI3K or p53 may not predict response to combinatorial therapy with oxaliplatin and everolimus.

Thus, the power of our current study is that combination of a genomic based approach with *in vitro* and *in vivo* validation using a preclinical model of colorectal cancer to show that the combination of oxaliplatin and a mTOR inhibitor is synergistic can be considered in patients with CRC liver metastasis. Although our initial study focused on the mTOR pathway, further analysis of the gene set enrichment analysis (**Table 3**) offer other opportunities to look at combinatorial therapies such as oxaliplatin and a WNT inhibitor in the treatment of colorectal cancer liver metastasis.

## Conclusions

Our current studies have provided a blueprint for the use of a genomic based approach coupled with both *in vitro* and *in vivo* preclinical models to identify optimal combinations of cytotoxic chemotherapy and a biological agent. Specifically, the combination of oxaliplatin and a mTOR inhibitor (everolimus) can potentially be used in the treatment of colorectal cancer liver metastasis.

## Supporting Information

S1 Fig Unsupervised hierarchical clustering on 39 colorectal cancer liver metastasis samples were performed with oxaliplatin treated samples boxed. Unsupervised hierarchical clustering showed that oxaliplatin treatment did not influence the clustering of the 39 samples.
(TIF)

S1 Table Enriched/upregulated oncogenic signaling pathways in patients with colorectal cancer liver metastasis treated with oxaliplatin. (DOCX)

## Abbreviations

VEGF: vascular endothelial growth factor
EGFR: epithelial growth factor receptor
GSEA: gene set enrichment analysis
PDXs: Patient derived xenografts
PBS: phosphate buffered saline
NSLC: non-small cell lung cancer
