# Supplementary figures and images for: Activation of the mTOR Pathway by Oxaliplatin in the Treatment of Colorectal Cancer Liver Metastasis
Source: PLoS One. 2017 Jan 6;12(1):e0169439. doi: 10.1371/journal.pone.0169439 (PMC5218497; doi:10.1371/journal.pone.0169439)

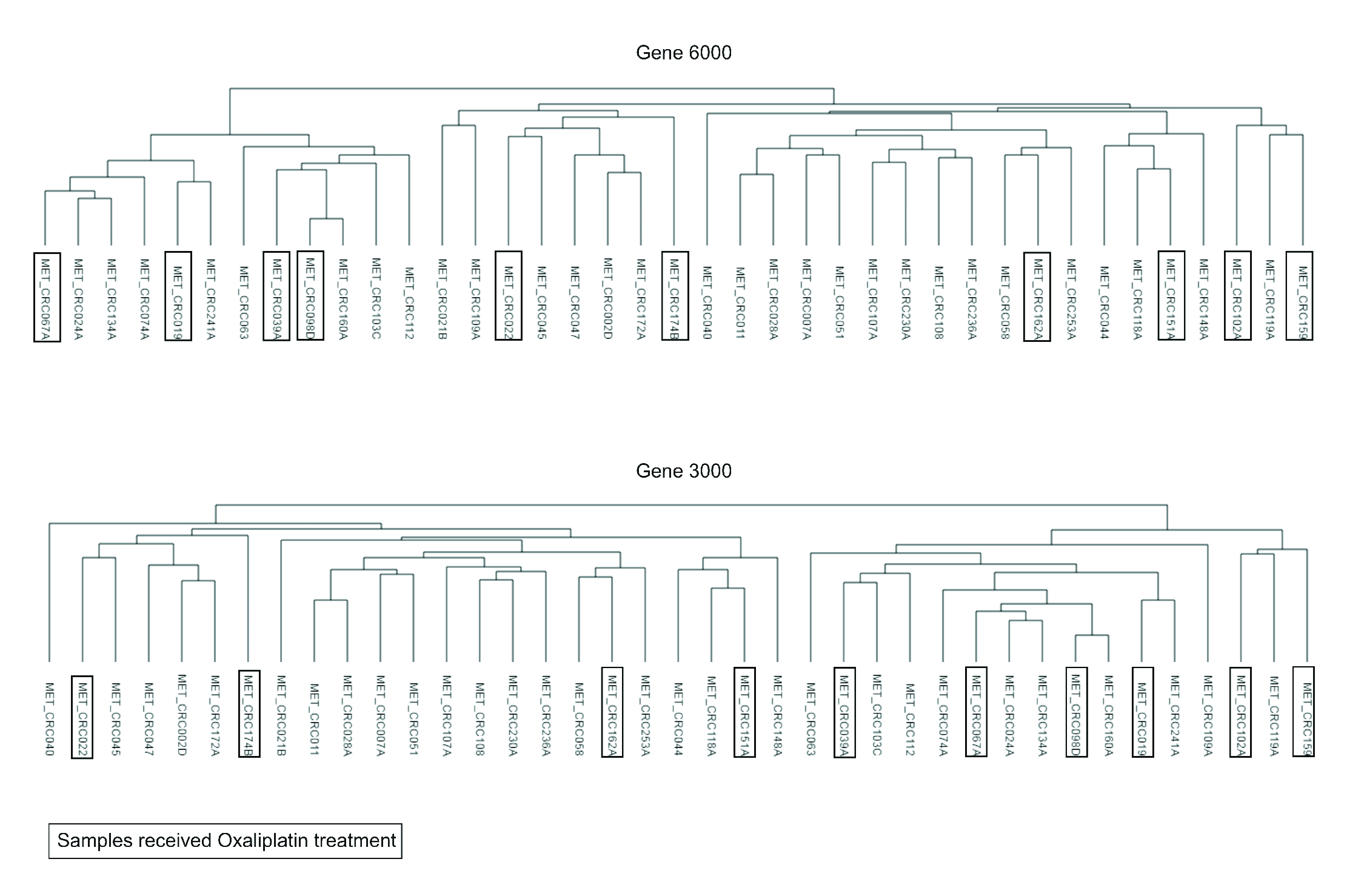

Supplement: S1 Fig — Unsupervised hierarchical clustering showed that oxaliplatin treatment did not influence the clustering of the 39 samples. (TIF) [file pone.0169439.s001.tif]
