# Supplementary material for: Activation of the mTOR Pathway by Oxaliplatin in the Treatment of Colorectal Cancer Liver Metastasis
Source: PLoS One. 2017 Jan 6;12(1):e0169439. doi: 10.1371/journal.pone.0169439 (PMC5218497; doi:10.1371/journal.pone.0169439)
Supplement: S1 Table — (DOCX) [file pone.0169439.s002.docx]

| **NAME** | **SIZE** | **ES** | **NES** | **NOM p-val** |
| --- | --- | --- | --- | --- |
| ESC_J1_UP_LATE.V1_DN | 156 | 0.45 | 1.61 | 0.00 |
| GCNP_SHH_UP_LATE.V1_DN | 155 | 0.45 | 1.60 | 0.00 |
| CRX_NRL_DN.V1_UP | 123 | 0.41 | 1.60 | 0.00 |
| CYCLIN_D1_KE_.V1_UP | 174 | 0.48 | 1.57 | 0.01 |
| BCAT.100_UP.V1_DN | 37 | 0.53 | 1.55 | 0.03 |
| KRAS.DF.V1_DN | 168 | 0.42 | 1.54 | 0.01 |
| ATF2_UP.V1_DN | 170 | 0.63 | 1.52 | 0.02 |
| PKCA_DN.V1_DN | 140 | 0.52 | 1.52 | 0.03 |
| E2F1_UP.V1_DN | 170 | 0.58 | 1.51 | 0.03 |
| ATF2_S_UP.V1_DN | 171 | 0.60 | 1.49 | 0.03 |
| HINATA_NFKB_IMMU_INF | 17 | 0.84 | 1.48 | 0.02 |
| PTEN_DN.V2_DN | 124 | 0.43 | 1.47 | 0.02 |
| DCA_UP.V1_DN | 155 | 0.41 | 1.47 | 0.03 |
| JNK_DN.V1_DN | 170 | 0.45 | 1.45 | 0.04 |
| CRX_DN.V1_DN | 122 | 0.49 | 1.44 | 0.07 |
| BCAT_GDS748_UP | 46 | 0.58 | 1.43 | 0.02 |
| WNT_UP.V1_UP | 160 | 0.44 | 1.43 | 0.03 |
| SNF5_DN.V1_DN | 146 | 0.50 | 1.43 | 0.05 |
| MTOR_UP.N4.V1_DN | 161 | 0.44 | 1.42 | 0.05 |
| NRL_DN.V1_UP | 124 | 0.35 | 1.42 | 0.02 |
| KRAS.AMP.LUNG_UP.V1_UP | 128 | 0.50 | 1.42 | 0.04 |
